# Forage, freedom of movement, and social interactions remain essential fundamentals for the welfare of high-level sport horses

**DOI:** 10.3389/fvets.2024.1504116

**Published:** 2024-11-20

**Authors:** Romane Phelipon, Noémie Hennes, Alice Ruet, Alexia Bret-Morel, Aleksandra Górecka-Bruzda, Léa Lansade

**Affiliations:** ^1^INRAE, CNRS, Université de Tours, PRC, Nouzilly, France; ^2^French Equestrian Federation, Lamotte-Beuvron, France; ^3^Department of Animal Behaviour and Welfare, Institute of Genetics and Animal Biotechnology, Polish Academy of Science, Magdalenka, Poland

**Keywords:** equestrian, horse management, athlete horses, horse living conditions, wellbeing indicators, stereotypies, equestrian competitions

## Abstract

Societal concerns for animal welfare extend to all domestic species, including high-level sport horses. The welfare of these horses, notably highlighted during the recent Olympics, has garnered significant public interest, prompting inquiries into their living conditions. Animal welfare studies have emphasised three key needs crucial to equine welfare: unlimited access to forage, freedom of movement, and social interactions with peers, commonly referred to as the “3Fs”—access to Forage, Freedom of movement, and interactions with Friend conspecifics. However, the feasibility and benefits of satisfying these needs specifically for sport horses remain unexplored. Indeed, they may face unique challenges such as high physical workload, extensive travel, limited time in their home stables, weight management, and high economic value necessitating careful handling. Consequently, restrictions on feeding, freedom of movement, and social contact are often deemed necessary. This field study aims to assess the actual level of implementation of welfare in high-level sport horses by evaluating body condition, injury risk, and behavioural welfare indicators in their home stable. To achieve this objective, the welfare of 56 high-level sport horses competing internationally was assessed using behavioural indicators of welfare through scan sampling (abnormal behaviours, i.e., stereotypies, aggression towards humans, withdrawn behaviour, and alert behaviours; positions of the ears in a backward position while foraging, watching behaviours, and through other Animal Welfare Indicators (AWIN) protocol measures). This study shows that there exists a large variability among horses regarding their access to the 3Fs, with some of them having a lot of restrictions and others not, meaning it is possible to respect them while competing at a high level. Second, we observed that the fewer restrictions the horses experience regarding the 3Fs while in their home stables, the better their welfare, as demonstrated by the indicators we assessed. These results undeniably support the fact that unrestricted access to forage, the ability to move freely outdoors, and the opportunity to interact socially with conspecifics are fundamental needs of horses that could be provided to horses, also to high-performance ones. It is therefore essential that solutions are put in place to ensure that these conditions are met.

## 1 Introduction

The welfare of high-level sport horses used in equestrian sports, and in particular the Olympic Games, is a growing societal issue that the International Equestrian Federation has recognised and responded to by setting up the Equine Ethics and Welfare Commission.^1^ There are many studies on the factors that promote the welfare of horses (1), including high-performance horses (2–4). However, high-level sport horses usually have very specific living and working conditions (2). They can undergo intensive daily training, travel extensively worldwide for competitions, spend limited time in their home stables, require careful weight management, and have high sentimental and economic value necessitating close monitoring, particularly to prevent injuries. Consequently, for their owners, restrictions on feeding, freedom of movement, and social interactions with conspecifics are often deemed necessary for the management of these conflicts, which arise between the demands of competition and the horse’s basic needs (5–8).

Restrictions of these natural needs negatively impact the welfare of horses (1). The three criteria related to these needs are called in the equine sector as the “3Fs”, a term first introduced by the equine behaviourist L. Fraser (Forage, Freedom, and Friends) and defined as groups of needs of horses that should be satisfied by horses.^2^ Unlimited forage is essential for the horse’s physiological needs as it can eat up to 16 h a day, providing fibres throughout the day (9). It has been observed that forage deprivation can rapidly cause health problems such as gastric ulcers (10) and metabolic changes (11). Food restriction can also lead to the development of abnormal behaviours, as suggested by studies showing a link between a higher prevalence of stereotypies and a limited quantity of hay in horses (4, 12). Freedom of movement, or the ability to move freely outside of working or training hours, is an essential criterion for a horse’s welfare. If this need is not met, it can affect their mental and physiological health (1, 4, 13–15). It has been shown that freedom of movement, whether in the paddock or pasture, reduces stereotypy (4, 16) and aggression towards humans (16). In addition, certain stress markers such as glucocorticoids decrease following locomotor activity (17), and oxytocin levels have been shown to increase during a daily paddock release period (4). Finally, as horses are social beings living in groups under natural conditions, long-term bonds are essential to them (18). Deprivation of social contact from a very young age affects horse welfare. The study by Heleski et al. (19) showed that young horses living individually in stalls exhibited more abnormal behaviours such as licking or chewing the stall than those living in groups. Another study showed that 67% of the young horses in the study developed at least one stereotypy when housed individually for the first time (20). Furthermore, being in an individual stall also has long-term effects, and the longer a horse is in an individual stall, the more likely it is to develop unresponsiveness to its environment (13). Finally, the restriction of social contact with other conspecifics also has a physiological impact, with individually housed horses showing impaired glucocorticoid levels as a marker of stress (21).

Despite the extensive literature on the impact of these 3Fs on welfare, studies are often limited to similar types of horses from equestrian centres, and sometimes racehorses (12, 22), with high-level sport horses rarely included (2–4). However, high-level sport horses have unique characteristics as described above. It is therefore necessary to study in more detail the possibility of assurance of 3Fs in these specific horses.

In horses, welfare can be evaluated through various indicators or protocols (23–29). One set of indicators concerns the prevalence of abnormal behaviours, such as stereotypies, that can be defined as a repetitive behaviour caused by frustration and repeated attempts at coping and may also be the result of a central nervous system dysfunction (30). Moreover, three behaviours have been identified as representative of poor welfare in horses: alert behaviours, withdrawn behaviours, and aggressiveness towards humans (31). The position of the ears while the horse eats is also an indicator of the horse’s welfare quality. In fact, it has been shown that horses feeding with their ears turned backward are also those showing a pessimist judgement bias (32) and is described as a reliable indicator of altered horse welfare (25).

Another indicator of a horse’s welfare is the behaviour of standing and scanning the environment. This behaviour is part of the horse’s normal behavioural repertoire as they typically spend between 6 and 10% of their time budget engaging in it (18). However, several studies show that when its prevalence is high, it could be a sign of altered welfare. Indeed, isolated mares exhibited more of this behaviour than mares confined to stalls or in pasture (33). Furthermore, it has been observed that the frequency of observation behaviour in horses is linked to chronic back pain (34). This makes this behaviour an interesting indicator of the horse’s welfare as it is a behaviour that can be easily observed and quantified. In addition to the behavioural indicators of welfare, we incorporated physical measures from the Animal Welfare Indicators protocol for horses (35), as the body condition score and alopecia.

The objectives of the present study were (1) to determine whether the 3Fs (Forage, Freedom, and Friends) are assured in high-level sport horses despite their specific use and (2) to investigate the relationships between categorical variables representative of each “F” and the expression of behavioural and physical indicators of poor welfare. Based on previous studies (1, 15, 22), we hypothesised that a proportion of elite sport horses would meet the 3Fs and that the 3Fs would remain essential for their welfare, notwithstanding their particular living conditions. We predicted that increased access to forage, freedom of movement, and social interactions with conspecifics in their usual stable environment would correlate positively with improved welfare indicators as lower prevalence of abnormal behaviours, reduced instances of backward ear positioning while foraging, and decreased vigilance behaviour. The study also examined the effects of unrestricted forage access on body condition scores and evaluated how increased freedom of movement and social contact influenced injury rates and skin alterations. This approach challenged the common practice of limiting these factors in high-performance horses, typically implemented to prevent injuries and manage weight (2, 7).

## 2 Materials and methods

### 2.1 Ethics statement

The authors compiled with the ARRIVE guidelines. The present study was approved by the Val de Loire Ethical Committee (CEEA VdL) and attributed a positive recommendation (authorisation number: CE19 – 2023-3110 – 2).

### 2.2 Animals studied

Fifty-six high-level sport horses from 13 French private stables were studied (12 mares, 30 geldings, and 14 stallions; mean age ± SD: 10.61 ± 2.69 years). During each visit to the 13 stables, a sample of between two and eight horses was studied over the course of the day. They were competing in show jumping and eventing at international levels, and 11 of them were considered for the French team for the 2024 Olympic Games. The horses were observed in their usual environment (their home stables) between March and September 2023, with the consent of their riders/owners and the French Equestrian Federation. The horses lived in boxes ranging from 13 square metres to 35 square metres (mean ± SD: 24.5 ± 5.7), and their bed material consisted of straw or shavings. Horses were all trained between 5 and 7 days a week, with each session lasting between 30 and 60 min, took part regularly in international competitions (some participating in more than 50 competitions a year), and had regular veterinary and osteopathic monitoring. Their living conditions are detailed below.

### 2.3 Forage access assessment

We evaluated whether the hay was distributed “*ad libitum*” or “rationed”. The criteria for unlimited access to hay were either a hay net system or portions large enough to leave sufficient hay when the next portion arrived. Based on this information, horses were grouped into two categories of the variable “access to forage” (F-forage): those with unlimited access to forage during the day and those with rationed forage.

### 2.4 Freedom of movement assessment

Freedom of movement assessment was determined according to the type of accommodation for each horse. The main type of housing (i.e., the housing where the horse spends the most hours during the day) for each horse was evaluated and included three possible levels of the variable “access to free movement” (F-movement): (1) over a 24 h day, the horse lives more outdoors than in the box: in this case, the horse was placed into the category “living more in pasture or paddock than box”; (2) over a 24 h day, the horse lives more in the box than into the paddock or pasture: in that case, the horse was placed in the category “mainly in box”; and (3) the horse lives in the box almost exclusively (goes outside in pasture or paddock for 1 h or less in a week): in this final case, the horse was placed in the category “box exclusively.”

### 2.5 Social interaction levels’ assessment

Social interaction assessment was determined according to the levels of possible contact with any other horses through their type of housing and evaluated for each horse by the observer during the visit of the stables. Three levels described in the Animal Welfare Assessment Protocol for horses (35) were used to categorise the level of possible social interaction for each horse. If the horse had at least the possibility to nibble and partly groom another horse, it was placed in the first category of the variable “access to social interaction” (F-social) variable labelled “tactile interaction”. This category included horses that could nibble each other through their box or paddock and those that could fully interact with other horses when placed together in a pasture or paddock. The second category included horses that could only sniff each other through the grids of their box or paddock and was labelled “possibility to sniff”. Finally, if the horse could only have visual contact with other horses, it was placed in the category labelled “visual interaction”. In this third category, horses can only see other horses from their box door or window, or from the paddock, but cannot sniff other horses muzzle to muzzle.

### 2.6 Abnormal behaviour ratio, backward ears ratio while foraging, and watching behaviour ratio

The evaluation of these behaviours was done using the scan sampling method over the course of a whole day by a single person (a PhD student of ethology) who had been previously trained in this method. This method previously described (36) consists of repeatedly observing the horse’s behaviour at regular intervals (here every 2 min). The observer walked discretely 2 m from the stall or paddock and noted the horse’s behaviour at a given moment. The observer should be silent to avoid being spotted by the horse being observed, to avoid any change in behaviour at the time of the observation. In the present study, 56 horses were observed over the course of a day from 9.20 a.m. and 5 p.m. The observer conducted a round every 2 min, moving from one horse to the next and noting the behaviour of each horse under observation (31). However, during the scan sampling observation periods, 16 horses were moved from the study for the purposes of undergoing training, undergoing veterinary check-ups, or otherwise, while they were observed. Consequently, they have been excluded from the subsequent analyses. As a result, 40 horses were observed for a total of 3,412 observations (85.3 ± 25.16).

The abnormal behaviour ratio was calculated from the observations of the scan sampling method. Based on the incidence of any of abnormal behaviours, the ratio included the following behaviours: stereotypies, aggressiveness towards humans, withdrawn, and hypervigilance behaviour. The ratio also included other abnormal behaviours such as gnawing on the bars of the box, gnawing on the feeder, door kicking, weaving, headshaking, crib-biting, licking on non-food, and tongue rolling. All abnormal behaviours are defined in the Supplementary Table S1. The abnormal behaviour ratio for each horse was calculated as follows: number of observations where the horse was displaying an abnormal behaviour divided by the total number of observations of the horse.

A previous study has shown that a chronic negative emotional state could be revealed by backward ears position in eating behaviours (32) and is a reliable indicator of compromised equine welfare (25). Therefore, backward ears while foraging has been used in the present study. Backward ears ratio while foraging was calculated from the observations of the scan sampling method and took into account all observations where the horse was foraging with its ears turned backward (apart during concentrate food distribution times and consumption). Positions of the ears are defined in Table 1. These observations were then divided by the total number of observations where the horse was foraging to obtain the backward ears ratio while foraging for each horse.

**Table 1:** Description of behaviours observed on horse.

Abnormal behaviour	Description
Stereotypies	Repeated behaviour which has no specific function or goal and does not vary (47). All stereotypies are described in the Supplementary Table S1.
Aggressiveness towards human	Threats or physical attacks directed towards a human (ears pinned backward sometimes with the mouth open) (31).
Withdrawn behaviour	Standing with eyes open, fixed gaze, few or no blinks, eyelids do not droop, and the horse do not respond to external sensory stimulus of its environment (31).
Alert behaviour	Remaining vigilant in position, with awareness and a raised neck, carefully surveying the environment, occasionally shifting either head or ears (48).
Other abnormal behaviours	Behaviours described in the Supplementary Table S1 as stereotypies but expressed once as gnawing on the box’s bar, gnawing on the feeder, weaving, headshaking, crib-biting, licking on non-food, and tongue rolling.

Ears position in eating behaviour	Description
Forward	The pinnae of both ears are directed forward.
On the side	The pinnae of both ears are directed on the side.
Backward	The pinnae of both ears are directed backward.
Asymmetric	The pinna of one ear is directed forward or to the side, while the other is directed backward.

Watching behaviour	Description
Watching behaviour	Standing still with the neck held horizontally or slightly raised, while the ears and neck remain mobile. The horse slowly surveys its surroundings by moving its head side to side or briefly looking at environmental stimuli occasionally (34).

Watching behaviour ratio also comes from observations of the scan sampling method. In the present study, we took into account all the behaviours in which the horse watched its environment (Table 1). The ratio was calculated by dividing the number of observations where the horse watched its environment by the total number of observations of the horse.

### 2.7 Physical measures

#### 2.7.1 Assessment of the body condition score

The body condition score was assessed by a single observer who had been previously trained in accordance with the guidelines of the French Horse protocol which is derived from the Animal Welfare Assessment Protocol for horses (35). The body condition score assessment was shown to be reliable, with assessors consistently scoring body condition after proper training (29). This assessment takes into account the evaluation of six body zones to obtain a final score for each horse.

The observer began with a general visual inspection of the horse. Then, six areas were specifically targeted: ribs, back of shoulder, withers, neck, tail attachment, and croup. For each area, a score from 1 (very thin/skeletal) to 5 (obese/overweight) was given according to the AWIN scoring scale. The score took into account the level of fat/muscle covering each area. The body condition score was then calculated from the scores for the six zones, with the different zones weighted as follows.


$$\begin{array}{l} \mathrm{Body}\;\mathrm{condition}\;\mathrm{score}=\left(\mathrm{rib}s^{’}\;\mathrm{score}\times 0.6\right)\\ \;+\left(\mathrm{back}\;\mathrm{of}\;\mathrm{the}\;\mathrm{shoulde}r^{’}s\;\mathrm{score}\times 0.15\right)\\ \;+\left(\mathrm{wither}s^{’}\;\mathrm{score}\times 0.10\right)+\left(neck^{’}s\;\mathrm{score}\times 0.15\right)\\ \;+\left(\mathrm{tail}\;\mathrm{attachmen}t^{’}s\;\mathrm{score}\times 0.05\right)+\left(\mathrm{crou}p^{’}s\;\mathrm{score}\times 0.005\right) \end{array}$$

#### 2.7.2 Assessment of the alopecia score

The alopecia score was evaluated by a single observer who had been trained in the Animal Welfare Assessment Protocol for horses beforehand (35). The alopecia took into account the total number of alopecia which could be defined as hairless spot or scar (35). There was no minimum size required to be taken into account, the observer looked at the whole body of the horse and noted all the alopecia and their localisation. Alopecia located on the horse’s equipment areas (e.g., noseband, headpiece, and girth passage) were not counted as they were not the result of access to forage, freedom of movement, and social interactions with other conspecifics.

### 2.8 Other measures included in the animal welfare assessment protocol for horses

We also recorded additional measures (Supplementary Table S2), from the Animal Welfare Assessment Protocol for horses (35) beyond those previously described. These measures were collected by the same observer on the same days as the previous observations. Thus, the following environmental measures were assessed: water availability, water cleanliness, sufficient quantity and cleanliness of bedding material, and adequate box dimensions. Furthermore, animal-based measures were evaluated, including integument alterations, absence of swollen joints, lameness and prolapse, hair coat condition, discharges, consistency of manure, breathing and absence of coughing, absence of horse grimace scale, state of the hoofs and lesions at the mouth corners, and a series of human–animal relationship tests.

### 2.9 Statistical analysis

All statistical analyses were performed using R software (R version 4.2.3) with a significance level set at a *p*-value of >0.05.

We used Fisher’s tests (*fisher.test* function) to check for potential collinearity between our variables of interest (access to forage: “F-forage,” access to freedom of movement: “F-movement,” access to social interaction: “F-social”). Significant associations were found between F-forage and F-movement (*p*-value <0.001), between F-movement and F-social (*p*-value <0.001), and between F-forage and F-social (*p*-value <0.001). A contingency table showing the number of horses for each variable was created to give an overview of the distribution of each group (Table 2). The purpose was to analyse the relationship between the F-variables and the abnormal behaviour ratio, the backward ears ratio while foraging, the alopecia score, and the watching behaviour ratio (Table 3). Given the collinearity between the F-variables, an initial stage of the analysis involved the joint examination of the F-variables using a multiple correspondence analysis (MCA). The MCA was used on the modalities of the F-variables, using the *MCA* function from the FactoMineR package (37). We then performed Spearman’s correlations (*cor.test* function) between the coordinates of individuals on dimension 1 and dimension 2 of the MCA and the behavioural (abnormal behaviour ratio, backward ears ratio while foraging, and watching behaviour ratio) and physical (alopecia score) measures.

**Table 2:** Contingency table presenting the relation between the access to forage (F-forage), freedom of movement (F-movement), and social interaction (F-social) of the horses.

F-movement				
		Box exclusively	Mainly in box	Pasture or paddock > box
F-forage	Limited	20	8	4
	Unlimited	2	14	8

F-social				
		Visual interaction	Possibility to sniff	Tactile interaction
F-forage	Limited	9	23	0
	Unlimited	6	10	8

F-movement				
		Box exclusively	Mainly in box	Pasture or paddock > box
F-social	Visual interaction	6	9	0
	Possibility to sniff	16	13	4
	Tactile interaction	0	0	8

**Table 3:** Results of Spearman’s correlation between the coordinates of Dim1 and Dim2 of the MCA and behavioural and physical variables.

		DIM1		DIM2	
Variables		Rho value	*p*-value	Rho value	*p*-value
Behavioural measure	Abnormal behaviour ratio	−0.56	**<0.001**	0.07	0.65
Behavioural measure	Backward ears ratio while foraging	**−0.46**	**0.0044**	**0.52**	**<0.001**
Behavioural measure	Watching behaviour ratio	−0.27	0.085	**0.42**	**0.006**
Physical measure	Alopecia score	**−0.48**	**<0.001**	**0.34**	**0.02**

Bold values indicate those with a *p*-value < 0.5.

Then, a more detailed analysis investigates each of the F-variables individually, while keeping in mind that these three variables are closely related. To compare the behavioural and physical measures as the abnormal behaviours, the backward ears ratio while foraging, the watching behaviour ratio, and the alopecia score between horses with limited access to forage and horses with unlimited access to forage, Mann–Whitney tests (*wilcox.test* function) have been used.

To compare whether the level of F-movement and F-social affected the behavioural measures (abnormal behaviour ratio, backward ears ratio while foraging, and watching behaviour ratio) and the physical measure (alopecia score), Kruskal–Wallis rank-sum tests (*kruskal.test* function) were used. Then, to find out between which modalities the significant differences were, *post-hoc* Dunn’s tests (*dunn.test* function) were performed with a Bonferroni correction.

The additional measures from the Animal Welfare Assessment Protocol for horses (35) are presented as descriptive analyses. Due to a lack of variability in the measures, no additional statistical analyses were performed on these data.

### 2.10 Data availability

Data generated and analysed during the study are available in the INRAE data repository from the following link: https://doi.org/10.57745/HLNFN3. Please contact R.P. for any request on the data availability.

## 3 Results

### 3.1 Descriptive analysis

In terms of access to forage, 57.1% of the horses studied were observed to have limited access, while 42.9% were observed to have unlimited access. Regarding the level of freedom of movement, 39.3% of the horses were living in box exclusively, 39.3% were living mainly in box, and 21.4% were living more in pasture or paddock than in the box. Concerning social interaction, 26.7% of horses had visual interaction with other horses, 58.9% had the possibility of sniffing other horses, and 14.2% could experience tactile interaction with other horses (Supplementary Table S2).

Out of 40 horses observed using the scan sampling method and regarding the abnormal behaviours, 20% performed at least one stereotypy, 20% showed aggressiveness towards humans, 2.5% alert posture, and no withdrawn posture was observed.

With regard to the environmental measures of the Animal Welfare Assessment Protocol for horses (35), it was observed that 100% of horses had access to clean water, 88.8% of horses had a sufficient quantity of bedding material, 100% had clean bedding material and adequate stall dimensions. For the animal-based variables, 100% of them did not show swollen joints, signs of hoof neglect, deep wound, lesions at mouth corners, prolapse, and ocular, vulva, or penis discharge. In addition, 100% showed a healthy hair coat condition, normal breathing, and an absence of pain grimace, and 97.9% had an optimal body condition score. Only 1.8% had a cough, 10% had a slight nasal discharge, and 5.5% of the manure had an abnormal consistency. At least one alopecia was found in 97.7% of the horses (median = 5.5[IQ1: 4, IQ3:13.25], min = 1, max = 30), 29.1% had at least one skin lesion (median = 0[IQ1: 0, IQ3:1], min = 1, max = 3), and 25% had at least a swelling (median = 0[IQ1: 0, IQ3:0.25], min = 1, max = 4). Finally, with regard to the human–animal relationship tests, 94.4% of horses show no negative signs for the avoidance distance test, as well as 89.3% for the voluntary animal approach test and 88.8% for the forced human approach test. All the data of the protocol are summarised in the Supplementary Table S2.

### 3.2 Relationships between the F-variables and the indicators of welfare

**Figure 1:**
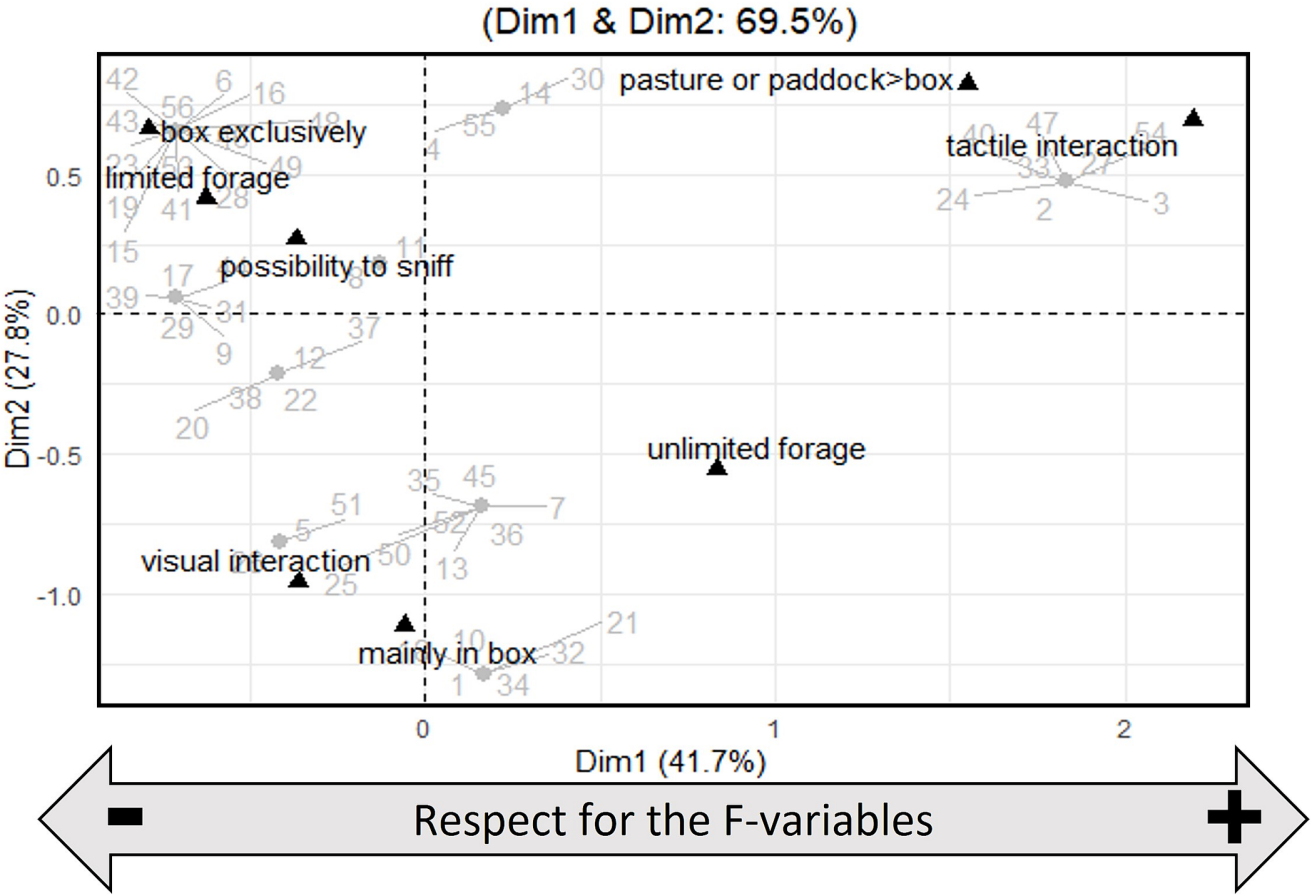
Multiple correspondence analysis (MCA) plot of the F-variables. The grey points and numbers correspond to the individuals.

Regarding the MCA results, the first dimension (Dim1) explained 41.7% of the overall variance, and the second dimension (Dim2) explained 27.7% of the overall variance (Figure 1). For Dim1, the F-forage was correlated at 0.52, the variable F-movement was correlated at 0.76, and the variable F-social was correlated at 0.80. Thus, the Dim1 was labelled “Respect for the F-variables”. For Dim2, the variable F-forage was correlated at 0.22, the variable F-movement was correlated at 0.80, and the variable F-social was correlated at 0.35. Thus, the Dim2 represents the weight of F-movement.

The coordinates on Dim1 were significantly and negatively correlated with the abnormal behaviour ratio, the backward ears ratio while foraging, and the alopecia score. It tends to be correlated with the watching behaviour ratio (Table 3). The coordinates on Dim2 were significantly and positively correlated with the backward ears ratio while foraging, watching behaviour ratio, and the alopecia score but not with the abnormal behaviour ratio (Table 3).

### 3.3 Effect of “forage access” categorisation (F-forage)

**Figure 2:**
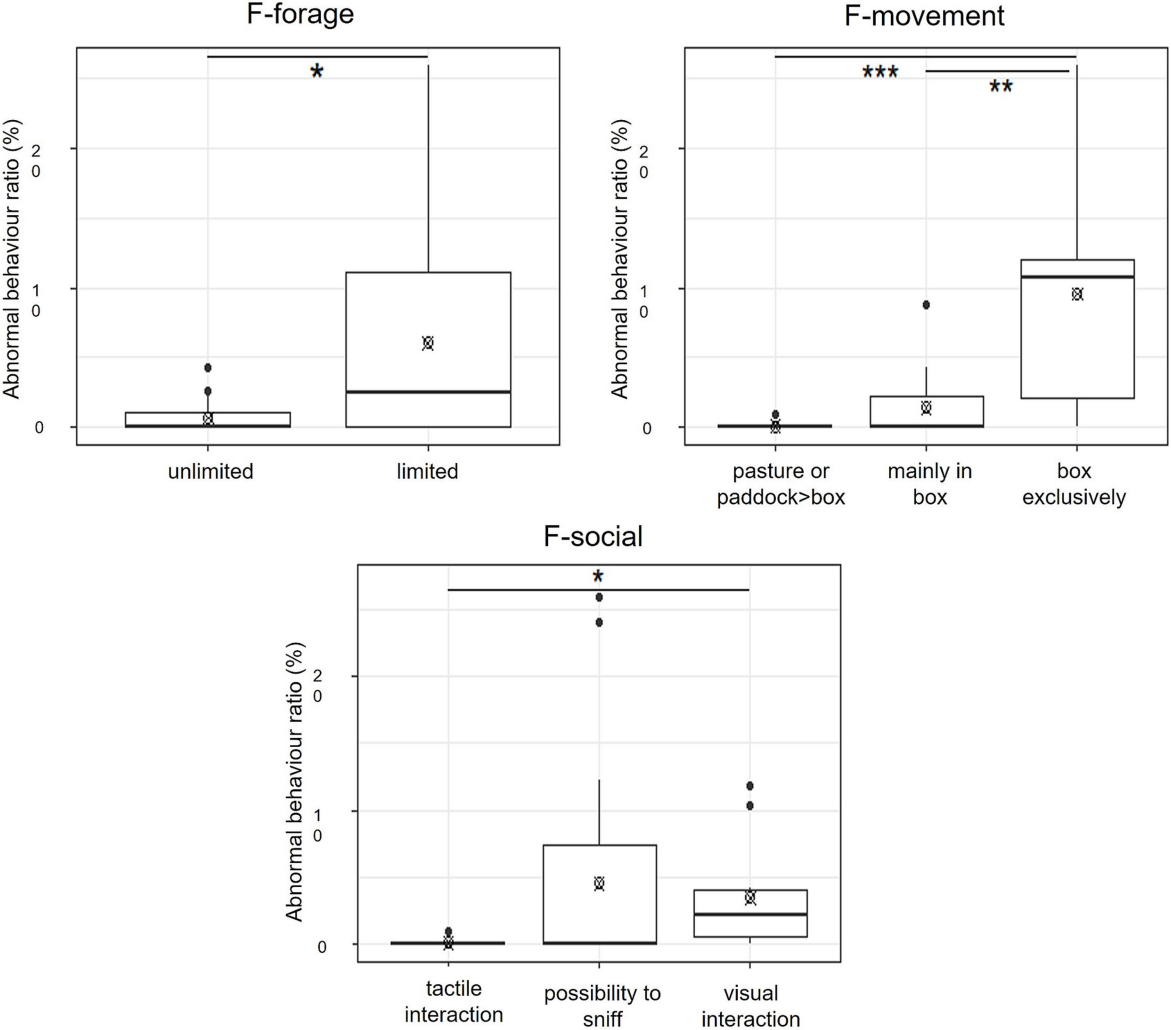
Median value, mean value (crossed circle), and interquartile ranges of the percentage of abnormal behaviour ratio depending on the F-forage: limited (*N* = 32) or unlimited (*N* = 24), on the F-movement: pasture or paddock > box (*N* = 12), mainly in box (*N* = 22), and in box exclusively (*N* = 22), and on the F-social: tactile interaction (*N* = 8), possibility to sniff (*N* = 33), and visual interaction (*N* = 15), * *p*-value <0.05, ** *p*-value <0.01, and *** *p*-value <0.001. The abnormal behaviour ratio was calculated as the number of abnormal behaviour observations/total observations.

Percentages of abnormal behaviour were significantly higher for horses with limited access to forage compared to horses with unlimited access to forage (Figure 2, Mann–Whitney test, W = 121.5, *p*-value = 0.021).

Percentages of ears backward while foraging were significantly higher for horses with limited access to forage (Mann–Whitney test, W = 246.5, *p*-value <0.001) compared to horses with unlimited access to forage (Table 4). Percentages of watching behaviour were higher for horses with limited access to forage (Mann–Whitney test, W = 275.5, *p*-value = 0.04) compared to those with unlimited access (Table 4). The alopecia score did not differ depending on the type of access to the forage (Mann–Whitney test, W = 300.5, *p*-value = 0.11, Table 4).

**Table 4:** Relationship between behavioural and physical measures according to the F-variables.

F-forage	Behavioural measures			Physical measure
	Abnormal behaviour ratio	Backward ears ratio while foraging	Watching behaviour ratio	Alopecia score
Limited forage	0 [IQ1: 0, IQ3: 4]^**a**^	0 [IQ1: 0, IQ3: 5]^**a**^	12.5 [IQ1: 7.47, IQ3:21.21]^**a**^	6 [IQ1: 4, IQ3: 13.5]
Unlimited forage	0 [IQ1: 0, IQ3: 3.75]	0 [IQ1: 0, IQ3: 5]	12.5 [IQ1: 7.62, IQ3: 20.6]	6 [IQ1: 4, IQ3: 13.5]

F-movement	Behavioural measures			Physical measure
	Abnormal behaviour ratio	Backward ears ratio while foraging	Watching behaviour ratio	Alopecia score
Pasture or paddock > box	0 [IQ1: 0, IQ3: 3.75]^**b**^	0 [IQ1: 0, IQ3: 5]	12.5 [IQ1: 7.62, IQ3: 20.6]	6 [IQ1: 4, IQ3: 13.5]^**b**^
Mainly in box	0 [IQ1: 0, IQ3: 3.88]^**b**^	0 [IQ1: 0, IQ3: 5]^**b**^	12.18 [IQ1: 7.54, IQ3: 19.62]^**b**^	5 [IQ1: 4, IQ3: 13]^**b**^
Box exclusively	0 [IQ1: 0, IQ3: 4.06]	0 [IQ1: 0, IQ3: 5]	12.82 [IQ1: 7.15, IQ3: 21.24]	7 [IQ1: 4, IQ3: 14]

F-social	Behavioural measures			Physical measure
	Abnormal behaviour ratio	Backward ears ratio while foraging	Watching behaviour ratio	Alopecia score
Visual interaction	0 [IQ1: 0, IQ3: 4]^**c**^	0 [IQ1: 0, IQ3: 5]	12.5 [IQ1: 7.47, IQ3: 20]	15 [IQ1: 3.75, IQ3: 13.25]
Possibility to sniff	0 [IQ1: 0, IQ3: 4]	0 [IQ1: 0, IQ3: 5]	12.5 [IQ1: 7.47, IQ3: 21.21]	6 [IQ1: 4, IQ3: 13.5]
Tactile interaction	0 [IQ1: 0, IQ3: 3.88]	0 [IQ1: 0, IQ3: 5]	12.5 [IQ1: 7.47, IQ3: 21.21]	5 [IQ1: 4, IQ3: 13.5]

For the F-forage part: Mann–Whitney test, median [Interquartile 1, Interquartile 3]. If followed by the upper case “a” (e.g., median [IQ1, IQ3]^**a**^), it indicates a significant difference with horses with unlimited access to forage. For the F-movement and the F-social parts: Kruskal–Wallis rank-sum tests, median [Interquartile 1, Interquartile 3]. If followed by the upper case “b” (e.g., median [IQ1, IQ3]^**b**^), it indicates a significant difference with horses living in box exclusively. If followed by the upper case “c” (e.g., median [IQ1, IQ3]^**c**^), it indicates a significant difference with horses that have the opportunity to fully interact with other horses.

### 3.4 Effect of “access to freedom of movement” categorisation (F-movement)

Percentages of abnormal behaviour differed depending on the access level of the F-movement variable (Kruskal–Wallis rank-sum test, *p*-value < 0.001). Horses housed in boxes exclusively showed a higher percentage of abnormal behaviour compared to horses living more in pasture or paddock than in box (Figure 2, Dunn’s test, *p*-value < 0.001) and horses living mainly in box (Figure 2, Dunn’s test, *p*-value = 0.005).

Holding the ears back while foraging percentages differed depending on the access level of the F-movement variable (Kruskal–Wallis rank-sum test, *p*-value < 0.001, Table 4). Horses living exclusively in the box had a higher percentage of backward ears while foraging than horses living mainly in the box (Dunn’s test, *p*-value <0.001, Table 4). There was a tendency showing that horses living more often on pasture or paddock than in box presented the ears back while foraging less often than horses living exclusively in box (Dunn’s test, *p*-value = 0.07, Table 4) but not than horses living mainly in box (Dunn’s test, *p*-value = 0.29, Table 4).

Watching percentages differed depending on the access level of the F-movement variable (Kruskal–Wallis rank-sum test, *p*-value = 0.01, Table 4). Horses living in box exclusively showed a higher percentage of watching behaviour than horses living mainly in box (Dunn’s test, *p*-value = 0.006, Table 4) but not with horses living more in pasture or paddock than in box (Dunn’s test, *p*-value = 0.23, Table 4). No difference in the watching behaviour percentage between horses living mainly in box and horses living more in pasture or paddock than in box has been shown (Dunn’s test, *p*-value = 0.30, Table 4).

Alopecia scores differed depending on the access level of the F-movement variable (Kruskal–Wallis rank-sum test, *p*-value = 0.001, Table 4). Horses living exclusively in box show a greater alopecia score than horses living mainly in box (Dunn’s test, *p*-value = 0.001, Table 4) and horses living more in pasture or paddock than box (Dunn’s test, *p*-value = 0.02, Table 4). No difference was found between horses living more in pasture or paddock than box and horses living mainly in box (Dunn’s test, *p*-value = 1, Table 4).

### 3.5 Effect of “access to social interaction” categorisation (F-social)

There is a tendency to show that the percentages of abnormal behaviour differed depending on the access level of the F-social variable (Kruskal–Wallis rank-sum test, *p*-value = 0.06). Horses that had only visual interaction with other horses showed a higher percentage of abnormal behaviour compared to horses that could experience tactile interaction with other horses (Figure 2, Dunn’s test, *p*-value = 0.031). No difference was found between horses that had the possibility to sniff other horses and horses that could have visual interaction with other horses (Figure 2, Dunn’s test, *p*-value = 0.38). No difference was found between horses that had the possibility to sniff other horses and horses that could have tactile interaction with other horses (Figure 2, Dunn’s test, *p*-value = 0.16).

No difference was found between the different levels of the F-social variable and behavioural measures as the percentage of backward ears while foraging (Kruskal–Wallis rank-sum test, *p*-value = 0.11, Table 4) or the percentage of watching behaviour (Kruskal–Wallis rank-sum test, *p*-value = 0.40, Table 4).

No difference was found either between the social interaction levels and the alopecia score (Kruskal–Wallis rank-sum test, *p*-value = 0.25, Table 4).

## 4 Discussion

High-level sport horses have very unique uses and living conditions. They are intensively trained from an early age to maintain optimum physical condition, travel extensively around the world to compete at the highest level, and are closely monitored by their vets and grooms to avoid injuries and optimise their weight. This study shows that there exists a large variability among horses regarding their access to forage, freedom of movement, and social contact, with some horses experiencing a lot of restrictions while other do not, meaning that it is possible to respect the needs of horses related to 3Fs while competing at a high level. Second, we observed that the fewer restrictions the horses experience regarding the 3Fs while in their home stables, the better their welfare is, as demonstrated by the indicators we assessed.

### 4.1 Applying the 3Fs is essential to the welfare of high-level sport horses

The significant correlation between dimension 1 of the ACM, which can be interpreted as a global indicator of respect for the 3Fs variables (access to forage, freedom of movement, and social interaction with conspecifics), and the abnormal behaviour ratio, the backward ears ratio while foraging, and the alopecia score shows that adherence to the 3Fs variables is strongly associated with improved welfare indicators in high-level sport horses. Specifically, horses that have greater access to these fundamental needs exhibit fewer abnormal behaviours, display more positive emotional states while eating, and even show better physical condition as evidenced by less alopecia. The detailed analysis of each of the 3Fs variables confirms this result and provides more specific insights into the effects of each of them, although it is important to keep in mind that these variables are interconnected.

Horses with unlimited access to forage exhibited little or no abnormal behaviour contrary to those with restricted access. Our findings support the main theories on equine feeding practices, which state that reducing the time spent eating affects the mental health of horses (38). In addition, horses with limited forage held the ears backward while foraging more compared than those with unlimited forage, which is a sign of a negative emotional state (32) and is uncommon to observe in naturalistic conditions (25). Finally, the high rate of watching behaviour in horses with limited forage could be explained by the fact that the horse’s main activity in terms of its time budget is eating (18), so not being able to perform this activity must be disruptive. The high rate of observation behaviour may also be attributed to the anticipation of the subsequent feed distribution. This is in accordance with the findings of the experimental setup, which indicated that this type of behaviour is more prevalent during the cue-arrival food phase (39). This raises the question of whether these horses, which observe their environment with greater frequency than others, at the expense of certain other behaviours, such as resting or eating, would perform redirected behaviours that could eventually develop into alert behaviour, which is an abnormal behaviour (Table 1). Unlimited forage is therefore essential for the welfare of high-level sport horses.

The second studied variable related to the freedom of movement. The more the horses had access to pasture or paddock, the less abnormal behaviour they exhibited. This finding is in line with a study of daily paddock access, in which horses were released into paddocks (4). Letting a horse out freely, even for a short time, can be very beneficial (1, 4, 7, 16, 19, 40). In fact, as little as 1 h a day, 5 days a week can already improve a horse’s welfare (4). Furthermore, horses that live exclusively in boxes are the ones that show more backward ears while foraging. This finding complements what was observed in the study where horses released into the paddock every day showed fewer backwards ears while foraging than those kept in boxes (4). These horses were also more observant than the others. One study showed that sport horses showed a higher frequency of standing alert behaviour when they were not turned out (8). In our study, we found that watching behaviour increased in horses kept exclusively in boxes compared to those living mainly in boxes. The positive impact of freedom of movement is evident even among high-level horses, despite their unique lifestyle.

Finally, our results show that high-level sport horses also need a high level of social contact. Our study revealed that horses that could only visually interact with other horses exhibited more abnormal behaviours than horses that could fully interact with other horses. Interestingly, although there are no significant differences, we found that horses that were only able to sniff other horses showed more abnormal behaviour than all other horses studied. In general, horses start by sniffing each other before engaging in social interaction as odours are a source of information about other horses (41). However, as only being able to smell a conspecific limits social interaction, we can question whether there is frustration in not being able to fully realise social interaction. Therefore, it would seem to be beneficial for the horse to be able to have tactile contact with other horses, even if this means partially nibbling each other across the boxes or paddock. Our results did not show that horses showed more ears backwards while foraging or more watching behaviour depending on the level of social interaction available, and this effect was more evident for abnormal behaviours.

The high-level sport horses whose living conditions best respect the needs for 3Fs (Forage, Freedom, and Friends) are those that show little to no signs of poor welfare. Compared to the other variables, social interaction has a slightly lesser impact. However, we believe this is related to the characteristics of our study population: Indeed, we have few horses with full access to conspecifics. With a different sample, with more horses that could have tactile interaction with conspecifics, this variable might have shown a greater impact. In conclusion to this first part, respect for the 3Fs is therefore essential for the welfare of these horses when they are in their stables.

### 4.2 Assuring the 3Fs is possible for high-level sport horses

Respect for 3Fs seems both necessary and possible. In fact, our study revealed that high-level stables already respect the 3Fs. Our study, based on voluntary sampling rather than probabilistic sampling, does not allow us to generalise the obtained proportions to the entire high-level equestrian sector. However, it does demonstrate that it is possible to reconcile adherence to 3Fs with competing at the highest level, including at the Olympic Games. In fact, some of the horses in our study whose living conditions complied with the 3Fs even won medals at the Paris 2024 Olympic Games. Moreover, the strong collinearity we found between the variables access to forage, freedom of movement, and social interaction with conspecifics shows that stables that respect one of these criteria also respect the other two. This suggests that it is possible to respect the 3Fs in the management of stables for high-level sport horses, yet this was not in evidence when talking to professionals in the equestrian world.

When it comes to forage, one of the arguments against unlimited access is often the fear that horses will gain too much fat. However, in our study, unlimited access to hay did not appear to have an effect on the body fat mass of elite sport horses, whereas hay restriction is known to have many negative consequences for the horse (10, 11, 42). This may be explained by the fact that high-level sport horses are involved in intensive sporting activity and are therefore less likely to gain weight. In the light of these findings, we therefore promote access to hay *ad libitum* for high-level sport horses.

One of the most interesting findings is the fact that horses living exclusively in boxes are the most injured (in terms of alopecia scores) compared with those living mainly in boxes and those living more in pastures or paddocks. This is a surprising result as it is generally thought that the opposite is true and that horses that are allowed to go out freely are the most likely to be injured. Indeed, this is one of the arguments for maintaining horses in boxes (43). However, one study demonstrated that, rather than age and sex being the primary factors influencing the incidence of injury, the breed had a more pronounced effect, thus suggesting that horses can be effectively maintained in groups comprising individuals of varying age and sex (44). Movement is essential for the welfare of the horses, and not providing this need can cause stress (4). It is therefore possible that the effect of living exclusively in a confined space could lead to an increase in the incidence of self-inflicted injury of these horses when they are expressing abnormal behaviours compared to horses that have the opportunity to move freely outside.

Our results also showed no difference in the body condition scores depending on the condition in which the horses are housed. Access to grass in the paddocks and pastures does not lead to greater weight gain. This finding is therefore an argument in favour of greater access to freedom of movement outside work, training, or exercise times for high-level sport horses.

Because of their value, sport horses are generally housed alone because of the risk of injury to each other. However, in our study, we found that the level of social contact had no impact on alopecia scores. This may be a reflection of the attention paid by the staff around these horses knowing the affinities between each horse. New systems are now possible to encourage this type of interaction, even for stallions that are usually isolated, such as converting boxes into social boxes (45). These devices enable horses to remain in individual accommodation but still have close tactile social contact with their neighbouring conspecifics when they want to.

Finally, the level of social interaction did not have an effect on the body condition score. As in some studies, isolation would lead to less feeding behaviour (20), a lower body condition scores for horses that only had visual interaction with others could have been observed. As the collinearity of our three variables was very strong, the horses that had only visual interaction were also the ones that were restricted in forage. In general, horses with limited access to forage are those that are fed high-concentrate diets (46), which may explain why there is no difference in their weight.

It should be noted that the present study was conducted with some limitations. As it was a field study, no reliability was conducted on the collected data due to time constraints at the private stables. In addition, the behaviour of the horses was observed on 1 day in their usual environment, which could have included the horse being in a box, paddock, or pasture. However, the findings of the study are nevertheless worthy of consideration. It is therefore necessary and possible to comply with the 3Fs as extensively as feasible with regard to high-level sport horses as they considerably enhance the level of welfare. The importance of the 3Fs must therefore be communicated as widely as possible to the institutions, organisations, and people involved in the equestrian industry including some key recommendations. It is therefore important to ensure that the fundamental needs of the sport horse, represented by the 3Fs, are met. This can be achieved by providing the horse with *ad libitum* forage throughout the day, allowing for daily extended periods of turnout outside of work, and ensuring that the horse has daily social interaction with its peers.

## Data Availability

The datasets presented in this study can be found in online repositories. The names of the repository/repositories and accession number(s) can be found at: https://doi.org/10.57745/HLNFN3, INRAE data repository.

## Footnotes

^1^ https://equinewellbeing.fei.org/

^2^ https://inside.fei.org/system/files/EEWBC%20Final%20Report%20to%20FEI%20Board_Updated%2014Nov23.pdf

## References

[1] Krueger K Esch L Farmer K Marr I. Basic needs in horses?—a literature review. Animals. (2021) 11:1–16. doi: 10.3390/ani11061798 PMC8235049 34208615

[2] Sauer FJ Hermann M Ramseyer A Burger D Riemer S Gerber V. Effects of breed, management and personality on cortisol reactivity in sport horses. PLoS One. (2019) 14:1–19. doi: 10.1371/journal.pone.0221794 PMC6886778 31790402

[3] Dalla Costa E Dai F Lebelt D Scholz P Barbieri S Canali E. Initial outcomes of a harmonized approach to collect welfare data in sport and leisure horses. Animal. (2017) 11:254–60. doi: 10.1017/S1751731116001452, PMID: 27406177

[4] Lesimple C Reverchon-Billot L Galloux P Stomp M Boichot L Coste C. Free movement: a key for welfare improvement in sport horses? Appl Anim Behav Sci. (2020):225. doi: 10.1016/j.applanim.2020.104972

[5] Furtado T Preshaw L Hockenhull J Wathan J Douglas J Horseman S. How happy are equine athletes? Stakeholder perceptions of equine welfare issues associated with equestrian sport. Animals. (2021) 11:1–18. doi: 10.3390/ani11113228 PMC8614509 34827960

[6] Krupa W Topczewska J Garbiec A Karpiński M. Is the welfare of sport horses assured by modern management practices? Anim Sci Genet. (2022) 18:57–77. doi: 10.5604/01.3001.0015.8108

[7] Werhahn H Hessel EF Van den Weghe HFA. Competition horses housed in single stalls (II): effects of free exercise on the behavior in the stable, the behavior during training, and the degree of stress. J Equine Vet. (2012) 32:22–31. doi: 10.1016/j.jevs.2011.06.009

[8] Werhahn H Hessel EF Schulze H Van den Weghe HFA. Temporary turnout for free exercise in groups: effects on the behavior of competition horses housed in single stalls. J Equine Vet Sci. (2011) 31:417–25. doi: 10.1016/j.jevs.2011.01.006

[9] Duncan P. Horses and Grasses. The Nutritional Ecology of Equids and Their Impact on the Camargue. New York, NY: Springer-Verlag (2012).

[10] Murray MJ Eichorn ES. Effects of intermittent feed deprivation, intermittent feed deprivation with ranitidine administration, and stall confinement with ad libitum access to hay on gastric ulceration in horses. Am J Vet Res. (1996) 57:1599–603. doi: 10.2460/ajvr.1996.57.11.1599, PMID: 8915437

[11] Di Filippo PA Duarte BR Albernaz AP Quirino CR. Effects of feed deprivation on physical and blood parameters of horses. Braz J Vet Med. (2021) 43:1–10. doi: 10.29374/2527-2179.bjvm000321 PMC9179197 35749104

[12] Mcgreevy PD Cripps PJ French NP Green LE Nicol CJ. Management factors associated with stereotypic and redirected behaviour in the thoroughbred horse. Equine Vet J. (1995) 27:86–91. doi: 10.1111/j.2042-3306.1995.tb03041.x, PMID: 7607155

[13] Ruet A Lemarchand J Parias C Mach N Moisan MP Foury A. Housing horses in individual boxes is a challenge with regard to welfare. Animals. (2019) 9:1–19. doi: 10.3390/ani9090621 PMC6770668 31466327

[14] Kádár R Maros K Drégelyi Z Szedenik Á Lukácsi A Pesti A. Incidence of compulsive behavior (stereotypies/abnormal repetitive behaviors) in populations of sport and race horses in Hungary. J Vet Behav. (2023) 61:37–49. doi: 10.1016/j.jveb.2023.01.003

[15] Bachmann I Audigé L Stauffacher M. Risk factors associated with behavioural disorders of crib-biting, weaving and box-walking in Swiss horses. Equine Vet J. (2003) 35:158–63. doi: 10.2746/042516403776114216, PMID: 12638792

[16] Ruet A Arnould C Levray J Lemarchand J Mach N Moisan MP. Effects of a temporary period on pasture on the welfare state of horses housed in individual boxes. Appl Anim Behav Sci. (2020):228. doi: 10.1016/j.applanim.2020.105027

[17] Hoffmann G Bockisch FJ Kreimeier P. Einfluss des haltungssystems auf die bewegungsaktivität und stressbelastung bei pferden in auslaufhaltungssystemen. Landbauforschung Volkenrode. (2009) 59:105–11.

[18] Waring GH. The behavioral traits and adaptations of domestic and wild horses, including ponies. New Jersey: Noyes Publications (1983).

[19] Heleski CR Shelle AC Nielsen BD Zanella AJ. Influence of housing on weanling horse behavior and subsequent welfare. Appl Anim Behav Sci. (2002) 78:291–302. doi: 10.1016/S0168-1591(02)00108-9

[20] Visser EK Ellis AD Van Reenen CG. The effect of two different housing conditions on the welfare of young horses stabled for the first time. Appl Anim Behav Sci. (2008) 114:521–33. doi: 10.1016/j.applanim.2008.03.003

[21] Yarnell K Hall C Royle C Walker SL. Domesticated horses differ in their behavioural and physiological responses to isolated and group housing. Physiol Behav. (2015) 143:51–7. doi: 10.1016/j.physbeh.2015.02.040 25725117

[22] Redbo I Redbo-Torstensson P Ödberg FO Hedendahl A Holm J. Factors affecting behavioural disturbances in race-horses. Anim Sci. (1998) 66:475–81. doi: 10.1017/S1357729800009644

[23] Dalla Costa E Minero M Lebelt D Stucke D Canali E Leach MC. Development of the horse grimace scale (HGS) as a pain assessment tool in horses undergoing routine castration. Hillman E, editor. PLoS one. (2014) 9:e92281. doi: 10.1371/journal.pone.0092281 24647606 PMC3960217

[24] Dalla Costa E Dai F Lebelt D Scholz P Barbieri S Canali E. Welfare assessment of horses: the AWIN approach. Anim Welf. (2016) 25:481–8. doi: 10.7120/09627286.25.4.481, PMID: 29303962

[25] Lesimple C. Indicators of horse welfare: state-of-the-art. Animals. (2020) 10:294. doi: 10.3390/ani10020294 32069888 PMC7070675

[26] Pritchard JC Lindberg AC Main DCJ Whay HR. Assessment of the welfare of working horses, mules and donkeys, using health and behaviour parameters. Prev Vet Med. (2005) 69:265–83. doi: 10.1016/j.prevetmed.2005.02.002 15907574

[27] Hausberger M Fureix C Lesimple C. Detecting horses’ sickness: in search of visible signs. Appl Anim Behav Sci. (2016) 175:41–9. doi: 10.1016/j.applanim.2015.09.005

[28] Mactaggart AG Phillips CJC. Validating a thoroughbred racehorse welfare index through horse behaviour and trainers’ reports of welfare issues in their horses. Animals. (2023) 13:282. doi: 10.3390/ani13020282, PMID: 36670822 PMC9855126

[29] Dalla Costa E Murray L Dai F Canali E Minero M. Equine on-farm welfare assessment: a review of animal-based indicators. Anim Welf. (2014) 23:323–41. doi: 10.7120/09627286.23.3.323, PMID: 32106531

[30] Mason G Clubb R Latham N Vickery S. Why and how should we use environmental enrichment to tackle stereotypic behaviour? Appl Anim Behav Sci. (2007) 102:163–88. doi: 10.1016/j.applanim.2006.05.041

[31] Ruet A Arnould C Lemarchand J Parias C Mach N Moisan MP. Horse welfare: a joint assessment of four categories of behavioural indicators using the AWIN protocol, scan sampling and surveys. Anim Welf. (2022) 31:455–66. doi: 10.7120/09627286.31.3.008

[32] Henry S Fureix C Rowberry R Bateson M Hausberger M. Do horses with poor welfare show “pessimistic” cognitive biases? Sci Nat. (2017) 104:8. doi: 10.1007/s00114-016-1429-1 28083632

[33] Mal ME Friend TH Lay DC Vogelsang SG Jenkins OC. Behavioral responses of mares to short-term confinement and social isolation. Appl Anim Behav Sci. (1991) 31:13–24.

[34] Rochais C Fureix C Lesimple C Hausberger M. Lower attention to daily environment: a novel cue for detecting chronic horses’ back pain? Sci Rep. (2016) 6:1–7. doi: 10.1038/srep20117 26823123 PMC4731760

[35] AWIN. AWIN welfare assessment protocol for horses. (2017); 1–80. Available at: https://air.unimi.it/retrieve/handle/2434/269097/384836/AWINProtocolHorses.pdf. (Accessed November 12, 2024).

[36] Altmann J. Observational study of behavior: sampling methods. Behaviour. (1974) 49:227–66. doi: 10.1163/156853974x00534 4597405

[37] Lê S Josse J Husson F. FactoMineR: an R package for multivariate analysis. J Stat Softw. (2008) 25:1–18. Available from: https://www.jstatsoft.org/index.php/jss/article/view/v025i01 (Accessed November 12, 2024).

[38] Sarrafchi A Blokhuis HJ. Equine stereotypic behaviors: causation, occurrence, and prevention. J Vet Behav. (2013) 8:386–94. doi: 10.1016/j.jveb.2013.04.068, PMID: 18267890

[39] Peters SM Bleijenberg EH van Dierendonck MC van der Harst JE Spruijt BM. Characterization of anticipatory behaviour in domesticated horses (*Equus caballus*). Appl Anim Behav Sci. (2012) 138:60–9. doi: 10.1016/j.applanim.2012.01.018

[40] Lansade L Valenchon M Foury A Neveux C Cole SW Layé S. Behavioral and transcriptomic fingerprints of an enriched environment in horses (*Equus caballus*). PLoS One. (2014) 9:1–19. doi: 10.1371/journal.pone.0114384 PMC4262392 25494179

[41] Guarneros M Sánchez-García O Martínez-Gómez M Arteaga L. The underexplored role of chemical communication in the domestic horse, *Equus caballus*. J Vet Behav. (2020) 38:89–95. doi: 10.1016/j.jveb.2020.05.008

[42] Brinkmann L Gerken M Riek A. Effect of long-term feed restriction on the health status and welfare of a robust horse breed, the Shetland pony (*Equus ferus caballus*). Res Vet Sci. (2013) 94:826–31. doi: 10.1016/j.rvsc.2012.10.010, PMID: 23141417

[43] Ross M Proudfoot K Merkies K Elsohaby I Mills M Macmillan K. Horse housing on Prince Edward Island, Canada: attitudes and experiences related to keeping horses outdoors and in groups. Animals. (2023) 13:1–15. doi: 10.3390/ani13020275 PMC9855179 36670815

[44] Keeling LJ Bøe KE Christensen JW Hyyppä S Jansson H Jørgensen GHM. Injury incidence, reactivity and ease of handling of horses kept in groups: a matched case control study in four Nordic countries. Appl Anim Behav Sci. (2016) 185:59–65. doi: 10.1016/j.applanim.2016.10.006

[45] Zollinger A Wyss C Bardou D Bachmann I. Social box: a new housing system increases social interactions among stallions. Animals. (2023) 13:1–17. doi: 10.3390/ani13081408 PMC10135302 37106974

[46] Thorne JB Goodwin D Kennedy MJ Davidson HPB Harris P. Foraging enrichment for individually housed horses: practicality and effects on behaviour. Appl Anim Behav Sci. (2005) 94:149–64. doi: 10.1016/j.applanim.2005.02.002

[47] Mason GJ. Stereotypies: a critical review. Anim Behav. (1991) 41:1015–37. doi: 10.1016/S0003-3472(05)80640-2, PMID: 39328347

[48] Pessoa GO Trigo P Mesquita Neto FD Lacreta Junior ACC Sousa TM Muniz JA. Comparative well-being of horses kept under total or partial confinement prior to employment for mounted patrols. Appl Anim Behav Sci. (2016) 184:51–8. doi: 10.1016/j.applanim.2016.08.014, PMID: 38102616

