# Supplementary material for: Forage, freedom of movement, and social interactions remain essential fundamentals for the welfare of high-level sport horses
Source: Front Vet Sci. 2024 Nov 20;11:1504116. doi: 10.3389/fvets.2024.1504116 (PMC11615640; doi:10.3389/fvets.2024.1504116)
Supplement: Supplementary file 1 [file Table_1.docx]

**Supplementary Material**

Table S1. Description of abnormal behaviours or stereotypies, if observed repeatedly during the study.

Behaviour Description

Stereotypies Repeated behaviour which has no specific function or goal and does not vary (1).

Aggressiveness toward human Threats or physical attacks directed toward a human (ears pinned backward sometimes with the mouth open) (2).

Withdrawn behaviour Standing with eyes open, fixed gaze, few or no blinks, eyelids don't droop and the horse do not response to external sensory stimulus of its environment (2).

Alert behaviour Remaining vigilant in position, with awareness and a raised neck, carefully surveying the environment, occasionally shifting either head or ears (3).

Gnawing on the box’s bar Gnawing at the bars of its box sliding its incisor teeth along the bar.

Gnawing on the feeder Gnawing at the feeder of its box sliding its incisor teeth along the feeder.

Weaving The horse sways its head and neck side to side, shifting its weight between its forelegs, occasionally coordinating movements with its hindquarters while remaining stationary (4).

Headshaking An atypical situation where the horse shakes its head without any apparent external trigger (5).

Crib-biting Seizing onto a stationary horizontal object (such as a fence, stall, or building structure) with the incisor teeth, simultaneously engaging the ventral neck muscles and pulling backward (4).

Licking on non-food Repetitive and prolonged action of the tongue on a non-food surface, typically observed on fences (6).

Tongue rolling Repetitive motion of turning and extending the tongue either outside or occasionally inside the open mouth (7).

Table S2. Results in percentage of horses satisfying the Animal Welfare Indicators protocol (AWIN, 2017) according to each indicator. The ‘-’ symbol means that the indicator could not be evaluated.

| **Good Feeding** | | | | | |
| --- | --- | --- | --- | --- | --- |
|  | | | **Yes (%)** | **No (%)** | **N** |
| Appropriate nutrition | Body condition score | | 97.96 | 2.04 | 49 |
| Absence of prolonged thirst | Water | Water availability | 100 | 0 | 54 |
|  |  | Water Cleanliness | 100 | 0 | 54 |
|  |  | Bucket test | - | - | - |
| **Good Housing** | | | | | |
| Comfort around resting | Bedding | Sufficient quantity of the bedding material | 88.89 | 11.11 | 54 |
|  |  | Cleanliness of the bedding material | 100 | 0 | 54 |
|  |  | Adequate box dimensions | 100 | 0 | 51 |
|  | Thermal Comfort | | - | - | - |
| Ease of movement | Frequency of exercise | Daily | 64.28 | 35.72 | 56 |
|  |  | Weekly (1 to 4 times a week) | 26.78 | 73.22 | 56 |
|  |  | Sometimes (less than 1 time a week) | 8.92 | 91.08 | 56 |
| **Good Health** | | | | | |
| Absence of injuries | Integument alterations | Absence of alopecia | 2.27 | 97.73 | 44 |
|  | | Absence of skin lesion | 70.83 | 29.17 | 48 |
|  | | Absence of deep wound | 100 | 0 | 48 |
|  | | Absence of swelling | 75 | 25 | 48 |
|  | Absence of swollen joints | | 100 | 0 | 47 |
|  | Absence of lameness | | - | - | - |
|  | Absence of prolapse | | 100 | 0 | 50 |
| Absence of disease | Healthy hair coat condition | | 100 | 0 | 53 |
|  | Absence of discharges | Nasal discharge | 90 | 10 | 50 |
|  | | Ocular discharge | 100 | 0 | 50 |
|  | | Vulva or penis discharge | 100 | 0 | 50 |
|  | Normal consistency of manure | | 94.44 | 5.56 | 36 |
|  | Normal breathing | | 100 | 0 | 49 |
|  | Absence of coughing | | 98.15 | 1.85 | 54 |
| Absence of pain and pain induced by management procedures | Absence of Horse Grimace Scale | | 100 | 0 | 50 |
|  | Absence of signs of hoof neglect | | 100 | 0 | 50 |
|  | Absence of lesions at mouth corners | | 100 | 0 | 48 |
| **Appropriate Behaviour** | | | | | |
| Expression of social behaviour | Social interaction | Possibility to nibble and partly groom | 14.29 | 85.71 | 56 |
|  | | Possibility to sniff other horses | 58.93 | 41.07 | 56 |
|  | | Possibility to have visual contact | 26.79 | 73.21 | 56 |
|  | | No possibility for visual of physical contact | 0 | 100 | 56 |
|  | Fear test |  | - | - | - |
| Good human-animal relationship | Human-animal relationship test | Absence of avoidance during the Avoidance Distance Test | 94.44 | 5.56 | 36 |
|  | | Absence of negative signs during the Voluntary Animal Approach Test | 89.36 | 10.64 | 47 |
|  | | Absence of negative signs during the Forced Human Approach Test | 88.89 | 11.11 | 45 |
|  | Positive emotional state | Qualitive Behaviour Assessment | - | - | - |

**References**

1. Mason GJ. Stereotypies: a critical review. Anim Behav. 1991;41(6):1015–37.

2. Ruet A, Arnould C, Lemarchand J, Parias C, Mach N, Moisan MP, et al. Horse welfare:A joint assessment of four categories of behavioural indicators using the AWIN protocol, scan sampling and surveys. Animal Welfare. 2022;31(4):455–66.

3. Pessoa GO, Trigo P, Mesquita Neto FD, Lacreta Junior ACC, Sousa TM, Muniz JA, et al. Comparative well-being of horses kept under total or partial confinement prior to employment for mounted patrols. Appl Anim Behav Sci. 2016;184:51–8.

4. Sarrafchi A, Blokhuis HJ. Equine stereotypic behaviors: Causation, occurrence, and prevention. Journal of Veterinary Behavior: Clinical Applications and Research [Internet]. 2013;8(5):386–94. Available from: http://dx.doi.org/10.1016/j.jveb.2013.04.068

5. Lane JG, Mair TS. Observations on headshaking in the horse. Equine Vet J. 1987;19(4):331–6.

6. Fernandez LT, Bashaw MJ, Sartor RL, Bouwens NR, Maki TS. Tongue twisters: Feeding enrichment to reduce oral stereotypy in giraffe. Zoo Biol. 2008;27(3):200–12.

7. Sun F, Zhao Q, Chen X, Zhao G, Gu X. Physiological Indicators and Production Performance of Dairy Cows With Tongue Rolling Stereotyped Behavior. Front Vet Sci. 2022;9(February):1–13.
